# The role of biomass burning states in light absorption enhancement of carbonaceous aerosols

**DOI:** 10.1038/s41598-020-69611-w

**Published:** 2020-07-30

**Authors:** Yu Wu, Tianhai Cheng, Xiaole Pan, Lijuan Zheng, Shuaiyi Shi, Hang Liu

**Affiliations:** 1 State Key Laboratory of Remote Sensing Science, Aerospace Information Research Institute, Chinese Academy of Sciences, No.9 Dengzhuangnan Road, Haidian District, Beijing, 100094 China; 2 State Key Laboratory of Atmospheric Boundary Layer Physics and Atmospheric Chemistry, Institute of Atmospheric Physics, Chinese Academy of Sciences, No.40 Huayanli, Chaoyang District, Beijing, 100029 China; 3 Land Satellite Remote Sensing Application Center, Ministry of Natural Resources of China, No.1 Baishengcun, Haidian District, Beijing, 100048 China

**Keywords:** Climate and Earth system modelling, Environmental impact

## Abstract

Carbonaceous aerosols, which are emitted from biomass burning, significantly contribute to the Earth’s radiation balance. Radiative forcing caused by biomass burning has been poorly qualified, which is largely attributed to uncertain absorption enhancement values (*E*_*abs*_) of black carbon (BC) aerosols. Laboratory measurements and theoretical modelling indicate a significant value of *E*_*abs*_; but this enhancement is observed to be negligible in the ambient environment, implying that models may overestimate global warming due to BC. Here, we present an aggregate model integrating BC aerosol ensembles with different morphologies and mixing states and report a quantitative analysis of the BC *E*_*abs*_ from different combustion states during biomass burning. We show that the BC *E*_*abs*_ produced by flaming combustion may be up to two times more than those produced by smouldering combustion, suggesting that the particle morphology and mixing state of freshly emitted BC aerosols is an important source of the contrasting values of *E*_*abs*_. The particle morphology of freshly emitted BC aerosols is widely assumed to be bare in models, which is rare in the ambient environment and leads to small estimates of *E*_*abs*_ by field observations. We conclude that the exact description of freshly emitted carbonaceous aerosols plays an important role in constraining aerosol radiative forcing.

## Introduction

Black carbon (BC) aerosols emitted from biomass burning are among the largest sources of uncertainty in assessments of positive radiative forcing and play an important role in global and regional climate change. Quantification of the warming caused by black carbon aerosols explicitly depends on the magnitude of the absorption enhancement (*E*_*abs*_) by mixing co-emitted weakly absorbing components through the so-called ‘lens effect’^1,2^. Previous theoretical modelling and laboratory measurements of BC aerosols have shown a significant enhancement up to a factor of ~ 3.5^3,4^. However, this enhancement is considered to be negligible (at ~ 1.06) by some field observations^5^, thereby causing a confusion regarding the parameterizations of BC absorption and thus leading to large uncertainties regarding the aerosol radiative forcing^6,7^. A possible source of controversy is that the measured particles may not be fully aged by coating with large non-BC material in the ambient environment, leading to underestimates of the BC absorption enhancements^8–10^. However, the effect of biomass burning states on BC absorption enhancements has not been qualified, and the bare particle morphology that is commonly assumed in modelling based on laboratory studies of freshly emitted BC aerosols may not be predominant under ambient conditions. Thus, the BC aerosols freshly emitted by different biomass burning states may be a crucial error source for estimating BC absorption enhancements.

In this study, we report a quantitative analysis of BC *E*_*abs*_ for different combustion states of biomass burning. The absorption properties of BC aerosols are qualified using a state-of-the-art theoretical model that considers more realistic morphologies of single particles^11^, and this aggregate model uses the superposition T-matrix method first developed for the BC aerosol ensembles. The modelled results for the dependence of BC *E*_*abs*_ on aging states show favourable agreement with previous measurements^12^, rather than the commonly used core–shell model with the Mie method. The distributions of particle size and mixing states are measured in the smouldering-dominated and flaming-dominated combustion states for the absorption calculations of carbonaceous aerosol ensembles^13^. Our simulated results indicate that the values of *E*_*abs*_ produced by flaming combustion are significantly larger than those produced by smouldering combustion, suggesting that the exact description of the particle morphology and mixing states of carbonaceous aerosols freshly emitted from biomass burning may play key roles in reducing the discrepancies of BC *E*_*abs*_ between field observations and theoretical modelling. Moreover, the effects of type and humidity of the biomass on BC *E*_*abs*_ are also investigated.

## Materials and methods

### Distributions of the particle size and mixing states in different combustion states

The biomass burning experiments, were performed in a combustion chamber in a laboratory environment and were conducted using dry wheat straw, wet wheat straw and dry rapeseed plants, as shown in Figure S1. Eighteen samples were directly burned in the chamber (referred to as “dry”), and four samples (referred to as “wet”) were placed in humid conditions (RH > 99%) for 30 min. A 50 cm long, 1/4-in. flexible conductive silicone tube was used for aerosol sampling, and a polytetrafluoroethylene (PTFE) tube was used for gas sampling. The residence time was very short (~ 6 s) to minimize the aging of the aerosols in the tube^13,14^. Most carbon substances are converted to carbon dioxide and BC particles in flaming-dominated combustion, while further smoldering-dominated combustion predominantly emits carbon monoxide and organics. The flaming and smoldering combustion stages were classified using the modified combustion efficiency (MCE), which was calculated from the fire-integrated excess CO and CO_2_ mixing ratios, relative to their background values. It is defined as ${{\Delta {\text{CO}}_{2} } / {\left( {\Delta {\text{CO}}_{2} + \Delta {\text{CO}}} \right)}}$, indicating different combustion states in biomass burning. An MCE value > 0.95 is normally regarded as flaming-dominant combustion, whereas an MCE value < 0.9 represents the smoldering-dominant combustion^15,16^. The mixing ratio of CO_2_ and CO was measured using a Li-7000 CO_2_ analyzer (Li-COR Inc.) and an ultrafast CO analyzer (model AL5002, Aero-Laser GmbH). A single particle soot photometer (SP2, Droplet Measurement Technologies Inc.) was used to measure the size distribution and shell-core ratio of BC. The distributions of the particle size and mixing states are measured in the smoldering-dominated and flaming-dominated combustion states. The commonly used shell-core ratio (S/C, or *F*_*sc*_ = *D*_*p*_/*D*_*c*_) is defined as the ratio of the volume-equivalent diameter of the shell (entire particle, *D*_*p*_) to the core (*D*_*c*_) particles, indicating the coating states of the carbonaceous aerosols. Microscopy observations have suggested that smoldering-dominated combustion leads to a smaller BC core and thicker non-BC coating. Thus, it tends to lead to a compact fractal aggregated BC structure^17–19^.

For different combustion stages, the mass size distribution of BC ensembles and the S/C ratio distribution of BC particles with fixed sizes were measured. The total masses of BC-containing particles (*M*_*p*_) are described by the lognormal functions of the mass equivalent diameter (MED) and the standard deviation (*σ*). The peak of MED (*D*_*m*_) is 215 nm for typical flaming-dominated combustion and 152 nm for typical smoldering-dominant combustion. The detailed parameters of the samples are shown in Table S1.

1 $$ M_{p} = \frac{1}{{D_{p} \sigma \sqrt {2\pi } }}e^{{ - \frac{{\left( {\ln D_{p} - \ln D_{m} } \right)^{2} }}{{2\sigma^{2} }}}}$$

Smoldering-dominated combustion tends to produce more thickly coated BC particles than flaming-dominated combustion. In this study, the S/C ratio distributions are described by Gaussian functions. When the MED of BC particles is 200 ± 10 nm, the measured peak values of S/C ratio (*F*_*m*_) are 1.18 for the flaming-dominated stage and 1.34 for smoldering-dominated stage.

2 $$ N\left( {F_{sc} } \right) = \frac{1}{{F_{sc} \sigma \sqrt {2\pi } }}e^{{ - \frac{{\left( {F_{sc} - F_{m} } \right)^{2} }}{{2\sigma^{2} }}}}$$

The variations of the measured S/C ratio can be described as a function of the MCE values. The peak of the MCE-dependent S/C ratio is fitted as follows:

3 $$ F_{m} = - 1.73 \times MCE + 2.86$$

Figure S2 shows the mass absorption cross sections (MAC) of the BC-containing aerosols was calculated using the distributions of the S/C ratio and peaks of the distributions. The absorption properties of carbonaceous aerosols were quantified using a state-of-the-art theoretical model considering more realistic particle morphologies (computational methods are introduced in the following section “Optical simulations by the aggregate model”). The deviations of the MAC between the poly-disperse and mono-disperse S/C ratios are limited to be less than 1.5%. Therefore, simulations of the BC-containing aerosols with the distributions of S/C ratios can be calculated by the peaks of the distributions.

### Physical properties of carbonaceous aerosols

The morphologies and compositions of black carbon aerosols depend on the types of fossil fuel or biomass source, burning process, and aging processes in the atmosphere^20,21^. Previous microscopy studies have indicated that freshly emitted BC particles consist of hundreds of small spherical primary particles combined into branched aggregates^22,23^. The construction and morphology of these particles can be described by the well-known fractal law^24,25^:

4 $$ Ns = k0\left( \frac{Rg}{a} \right)^{Df}$$

5 $$ R_{g}^{2} = \frac{1}{{N_{s} }}\sum\limits_{i = 1}^{{N_{s} }} {r_{i}^{2} }$$

where *N*_*s*_ is the number of monomers in the cluster, *a* is the mean radius of the monomers, *k*_*0*_ is the fractal prefactor, *D*_*f*_ is the fractal dimension, *R*_*g*_ is the radius of gyration representing the deviation of the overall aggregate radius in a cluster, and *r*_*i*_ is the distance from the *i*th monomer to the centre of the cluster. The S/C ratio is 1.0 for bare BC particles without mixing non-BC components. The fractal dimensions (*D*_*f*_) of bare and coated BC particles typically vary from 1.8 to 3.0 with a fractal prefactor of 1.2^18^. Previous studies showed that fractal dimensions of different types of BC particles ranged from 1.8 to 2.2 and increased during aging^19^. The sensitivity of fractal parameters on BC optical properties is investigated in Figure S3. Bond and Bergstrom reported the value of the mean radii of a BC monomer (*a)* to be in the range of 0.01–0.025 μm^24^. In field observations, the numbers of monomers (*N*_*s*_) have been observed to be in the range of 50–300, and may vary up to approximately 800^26^.

The refractive index of a BC component is assumed to be 1.95 + 0.79i in the visible and infrared range^24^. In the ambient environment, these refractive indices may vary across aerosol types. The real parts of the refractive indices of non-BC particles are assumed to be in the range of 1.4–1.6 and the imaginary part from 0 to 0.1^27^. These assumed refractive indices lie in the range of typical atmospheric aerosols, such as organics, sulfate, nitrate, dust, sea salt and brown carbon components^28–31^. In this study, the real refractive indices of the non-BC coatings were held constant at 1.55, and three values of 10^–2^, 10^–3^, and 10^–4^ for their imaginary refractive indices were assumed. Figure S4 indicates that the trends of the simulated values of the BC aerosols are consistent with the measurements by McMeeking et al.^12^, a refractive index of 1.55 + 10^−3^i for the non-BC component is suggested for the biomass burning absorption simulations. The sensitivity of the non-BC refractive indices to the BC optical properties is presented in Figure S5.

### Optical simulations by the aggregate model

Optical simulations were performed using the aggregate model parameterized by the complex particle morphology of BC at different aging scales. BC particles with bare, partly coated (thinly coated), partially encapsulated, and heavily coated states were modelled. Bare BC particles were modelled without any non-BC coating components using the diffusion limited aggregation (DLA) method, and aggregations of BC monomers were constructed with the given fractal parameters^32^. Partly coated BC particles were constructed by the aggregation of concentric core–shell spherical monomers. The partially encapsulated morphologies of BC-containing particles were represented by aggregated BC particles partially embedded in the host non-BC particle. For the heavily coated states, compact aggregated BC particles were internally mixed with large spherical non-BC particles, and all the BC monomers were inside the non-BC particles^33,34^. In this study, the fractal prefactor was assumed to be 1.2 and the fractal dimension was 1.8, 2.4, and 2.8 for the partly coated (including bare), partially encapsulated, and heavily coated states, respectively.

The morphologies of BC aerosols with different mixing states were modelled to initialize the superposition T-matrix method. This method uses numerically exact solution methods of Maxwell’s equations, which can be used to calculate the T-matrix descriptions of light scattering from the cluster with an appropriate superposition technique and thereby, analytically obtain the random-orientation cross sections and scattering matrices of these clusters. The superposition T-matrix method is applicable to a wide range of particle sizes and generates all of the scattering and absorption characteristics of the particles^35,36^. The random-orientation optical results were averaged using 10 random realizations of BC particles with the same morphological parameters to reflect the overall single scattering properties.

The cross sections of absorption (*C*_*abs*_) were calculated and integrated using the distributions of the particle size and S/C ratio.

6 $$ \left\langle {C_{abs} } \right\rangle = \int_{0}^{\infty } {\int_{1}^{\infty } {C_{abs} \left( {F_{sc} ,D_{p} } \right)\,N\left( {F_{sc} ,D_{p} } \right)\,dF_{sc} \,dD_{p} } }$$

7 $$ M_{p} = \frac{4}{3}\pi N\left( {D_{p} } \right)\left[ {\int_{1}^{\infty } {\left( {\rho_{BC} - \rho_{non - BC} } \right)\left( {\frac{{D_{p} }}{{{2}F_{sc} }}} \right)^{3} N\left( {F_{sc} } \right)dF_{sc} } + \rho_{non - BC} \left( {\frac{{D_{p} }}{2}} \right)^{3} } \right]$$

where *C*_*abs*_*(F*_*sc*_*,D*_*p*_*)* is the absorption cross sections of the individual BC particles. *N(F*_*sc*_*,D*_*p*_*)* is the number of the individual BC particles with fixed shell-core ratio (*F*_*sc*_) and particle size (*D*_*p*_). The mass density of BC (*ρ*_*BC*_) is assumed to be 1.8 g/cm^3^ according to the review of measurement by Bond and Bergstrom^24^, and the mass density of the non-BC components (*ρ*_*non-BC*_) is estimated to be 1.05 g/cm^3^^37^. The sensitivity of the density of the non-BC components in the individual BC-containing particles (1.0–1.2 g/m^3^) to the MAC of the BC aerosols at different combustion stages is investigated, as shown in Figure S6. $\left\langle {C_{abs} } \right\rangle$ is obtained by integration of the absorption cross sections of all BC particles with different sizes and S/C ratios. The single scattering albedo (SSA) is defined as $\left\langle {SSA} \right\rangle = \frac{{\left\langle {C_{{s{\text{ca}}}} } \right\rangle }}{{\left\langle {C_{abs} } \right\rangle { + }\left\langle {C_{sca} } \right\rangle }}$, where *C*_*abs*_ and *C*_*sca*_ are the cross sections for absorption and scattering, respectively.

The mass absorption cross sections of the BC aerosols were further normalized, which were defined as the cross section per unit mass of the particles. The normalization of the absorption cross sections is defined by the BC mass.

8 $$ MAC = \frac{{\left\langle {C_{abs} } \right\rangle }}{{\frac{4}{3}\pi \rho_{BC} \int_{0}^{\infty } {\int_{1}^{\infty } {\left( {\frac{{D_{p} }}{{2F_{sc} }}} \right)^{{3}} N\left( {F_{sc} ,D_{p} } \right)\,dF_{sc} dD_{p} } } }} $$

The *E*_*abs*_ is defined as the MAC between the BC aerosol ensembles including coated BC particles with the specific peak values of S/C ratio in the range of *F*_*m*_ > 1 and the BC aerosol ensembles are all bare BC particles with *F*_*m*_ = 1 ($E_{abs} = {{MAC_{{F_{m} > 1}} } \mathord{\left/ {\vphantom {{MAC_{{F_{m} > 1}} } {MAC_{{F_{m} = 1}} }}} \right. \kern-\nulldelimiterspace} {MAC_{{F_{m} = 1}} }}$).

The Ångström exponent (ÅE) over a wavelength interval $\left[ {\lambda_{1} ,\lambda_{2} } \right]$ is defined as

9 $$ A\mathop A\limits^{o} E = - \frac{{\ln \frac{{\left\langle {C_{abs} \left( {\lambda_{1} } \right)} \right\rangle }}{{\left\langle {C_{abs} \left( {\lambda_{2} } \right)} \right\rangle }}}}{{\ln \frac{{\lambda_{1} }}{{\lambda_{2} }}}} $$

Previous measurements indicated that the absorption ÅE values (AÅE) are near 1 (the theoretical value for black carbon) for AErosol RObotic NETwork-measured (AERONET) aerosol columns dominated by urban-industrial aerosol and larger AÅE values are observed for biomass burning aerosols^12^. The sensitivity of the incident wavelength on BC optical properties is presented in Figure S7, and the SSA and AÅE are shown in Figure S8.

The radiative properties of BC aerosols in climate models are commonly obtained based on the morphological simplification of spheres for the different mixing states, which are generally calculated using the Mie core–shell model. However, large discrepancies have been measured and simulated between the aggregates and the equivalent sphere approximations due to their complex morphologies, components and multiple scattering^38–40^.

The S/C ratio has been generally used in previous measurements of BC aerosols, because of the widely adopted Mie method. Therefore, in this study, the S/C ratio is applied for the comparisons of the aggregate model using the T-matrix method with the corresponding measurements by previous studies. In fact, the volume/mass fractions of BC and non-BC particles have also been used in previous studies^37^. For example, the S/C ratio can be directly calculated by the mass ratio of non-BC and BC components in individual BC-containing particles (*M*_*R*_ = *M*_*non-BC*_/*M*_*BC*_, non-BC mass *M*_*non-BC*_, and BC mass *M*_*BC*_) as *F*_*sc*_ in the following equation:

10 $$ F_{sc} = \sqrt[3]{{\frac{{M_{R} \rho_{BC} }}{{\rho_{non - BC} }}{ + 1}}} $$

In the core–shell model calculated using Mie theory, the volume-equivalent radius of BC (*R*_*BC*_) is related to their masses and their aggregated morphologies, according to the following equation:

11 $$ R_{BC} = \sqrt[3]{{\frac{{M_{BC} }}{{\frac{4}{3}\pi \rho_{BC} }}}} = \sqrt[3]{{N_{s} }}a $$

The thickness of the non-BC (*T*_*non-BC*_) shell is

12 $$ T_{non - BC} = \sqrt[3]{{\frac{3}{4\pi }\left( {\frac{{M_{BC} }}{{\rho_{BC} }} + \frac{{M_{non - BC} }}{{\rho_{non - BC} }}} \right)}} - R_{BC} $$

The mixing states of BC particles are quantified by the augmentation of the non-BC thickness. For bare BC particles, the thickness of the non-BC coating is zero. Thicker coating of non-BC components leads to larger values of the S/C ratio. Multi-scattering of fractal aggregated BC monomers in the individual BC-containing particles is not considered by the morphological simplifications of the single core–shell sphere model, and BC particles with inclusions, which are frequently found by microscopy measurements, may have significant effects on estimating the absorption enhancements of BC aerosols^41^.

## Results

**Figure 1:**
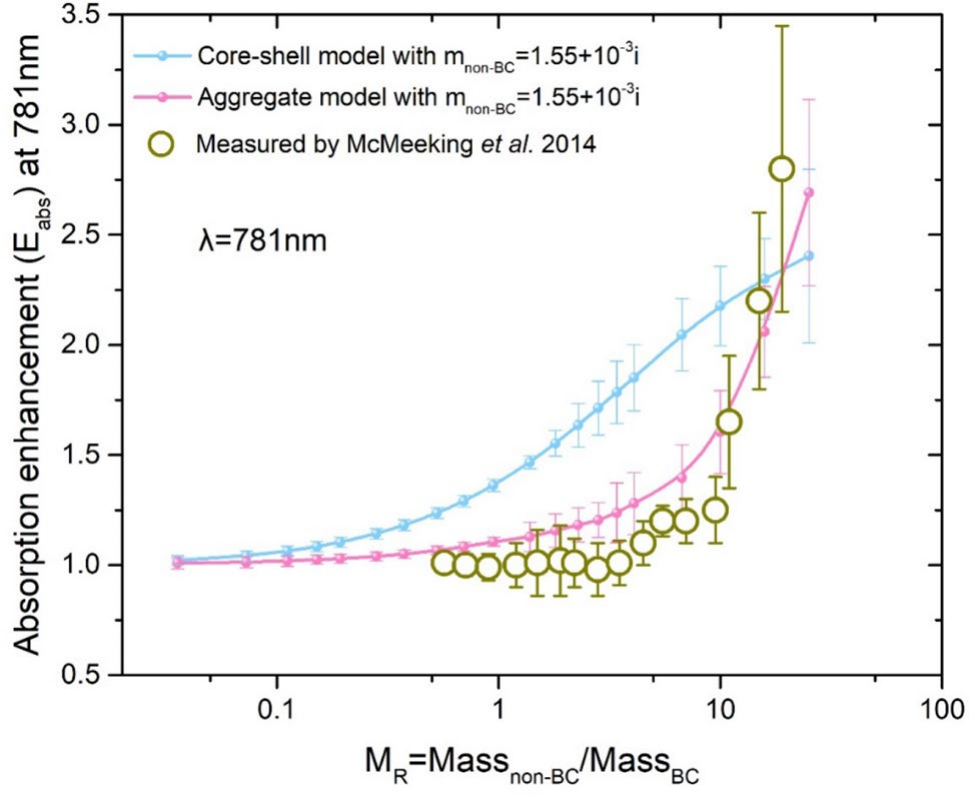
Comparison of the core–shell model by the Mie method (blue), the aggregate model by the T-matrix method (purple) and the measurements (dark yellow) by McMeeking et al.^12^ on *E*_*abs*_ at 781 nm. The refractive index of the BC particles is assumed to be 1.95 + 0.79i, and the refractive index of the non-BC component is assumed to be 1.55 + 10^−3^i.

Figure 1 indicates that the simulated light absorption enhancement (*E*_*abs*_) of BC aerosols, dependent upon aging states, shows a favourable agreement with previous measurements at 781 nm. The mass ratio of the non-BC and BC components is considered to be a key indicator of BC aging. The observations^12^ show that the simulations of the core–shell model calculated using Mie theory may introduce large deviations from the observations in general cases. In this study, the wavelength is 781 nm, and the refractive indices of the BC and non-BC particles are assumed to be 1.95 + 0.79i and 1.55 + 10^−3^i, respectively. As shown in Fig. 1, the deviations of the BC *E*_*abs*_ between the simulations by the Mie method (blue line/points) and the measurements by McMeeking et al. (dark yellow circles) are significant when the S/C ratio ranges from 1 to 10. In contrast, the presented aggregate model (purple line/points) is an improved fit for reproducing the BC *E*_*abs*_ over this range. Thus, the aggregate model using the T-matrix method with the BC complex particle morphology, rather than the core–shell model using the Mie method, is applied in the following sections for further investigations of BC aerosols freshly emitted from different biomass burning states.

**Figure 2:**
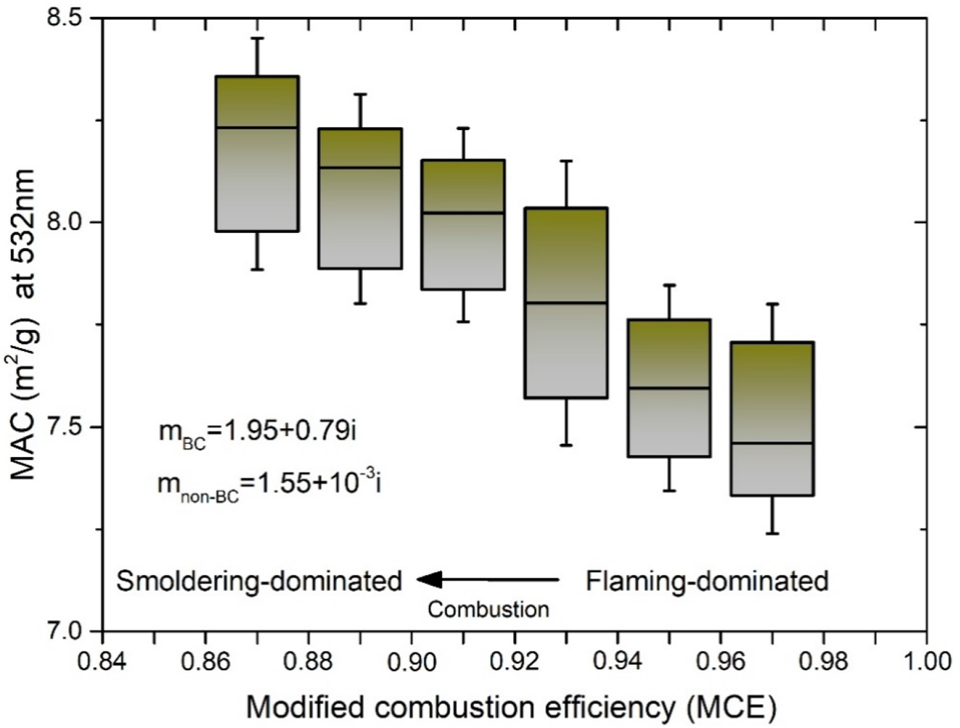
The mass absorption cross sections (MAC) of BC-containing aerosols at 532 nm predicted by the aggregate model is dependent on the modified combustion efficiency (MCE) of biomass burning, which indicates different combustion states. The MAC values of varied MCE are assembled with interval of 0.02 for each box, and the uncertainty is come from particle size, morphology, and mixing states of BC aerosols. Generally, flaming-dominated combustion corresponds to larger MCE (> 0.95) values, and smouldering-dominated combustion are corresponds to smaller MCE (< 0.9) values.

Figure 2 illustrates that the physical variations of carbonaceous aerosols caused by different combustion states may have a remarkable effect on light absorption. Burning experiments within laboratory combustion chambers were conducted using dry wheat straw, wet wheat straw, and dry rapeseed plants. The MCE-dependent distributions of the particle mass equivalent diameter (MED) and S/C ratio, measured assuming a BC density of 1.8 g/cm^3^, indicate different particle sizes, morphologies, and mixing states of the BC-containing aerosols arising from different combustion states. The atmospheric aging time was measured at less than 10 s, and thus, carbonaceous aerosols are considered to be freshly emitted from the burned biomass. The mass absorption cross sections (MAC) of the BC-containing aerosols at 532 nm were estimated using the aggregate model constrained by the particle morphology for different coating states: bare, partly coated (thinly coated), partially encapsulated (semi-embedded), and heavily coated (internally mixed)^11,18^. The refractive indices of the BC and non-BC particles were assumed to be 1.95 + 0.79i and 1.55 + 10^−3^i, respectively. Simulated MAC values of freshly emitted BC particles range between 7 and 8.5 at 532 nm depending on the MCE of the burned biomass, and this result agreed with recent measurements of controlled combustion^42,43^. As shown in Fig. 2, the MAC of the freshly emitted BC aerosols from the smouldering-dominated combustion states are ~ 1.09 times larger than those from flaming-dominated combustion at 532 nm, suggesting that smouldering combustion tends to produce more thickly coated BC particles than flaming combustion, thus intensifying the lens effect. The potential effects of brown carbon are also investigated, as shown in Figure S9. Different simulations for the dependence of the MCE on the imaginary parts of refractive indices of non-BC components were performed for brown carbon mixing with BC particles; the imaginary indices used were 0.001, 0.01, 0.02, and 0.1, while the real part was held constant at 1.55. The differences in the BC absorption for the different biomass burning states tended to become larger for the more absorbing non-BC components. The MAC of the freshly emitted BC aerosols from the smouldering-dominated combustion states are ~ 1.09, ~ 1.12, ~ 1.14, and ~ 1.21 times larger than those from flaming-dominated combustion at 532 nm when the refractive index imaginary parts of the non-BC components are 0.001, 0.01, 0.02, and 0.1, respectively.

**Figure 3:**
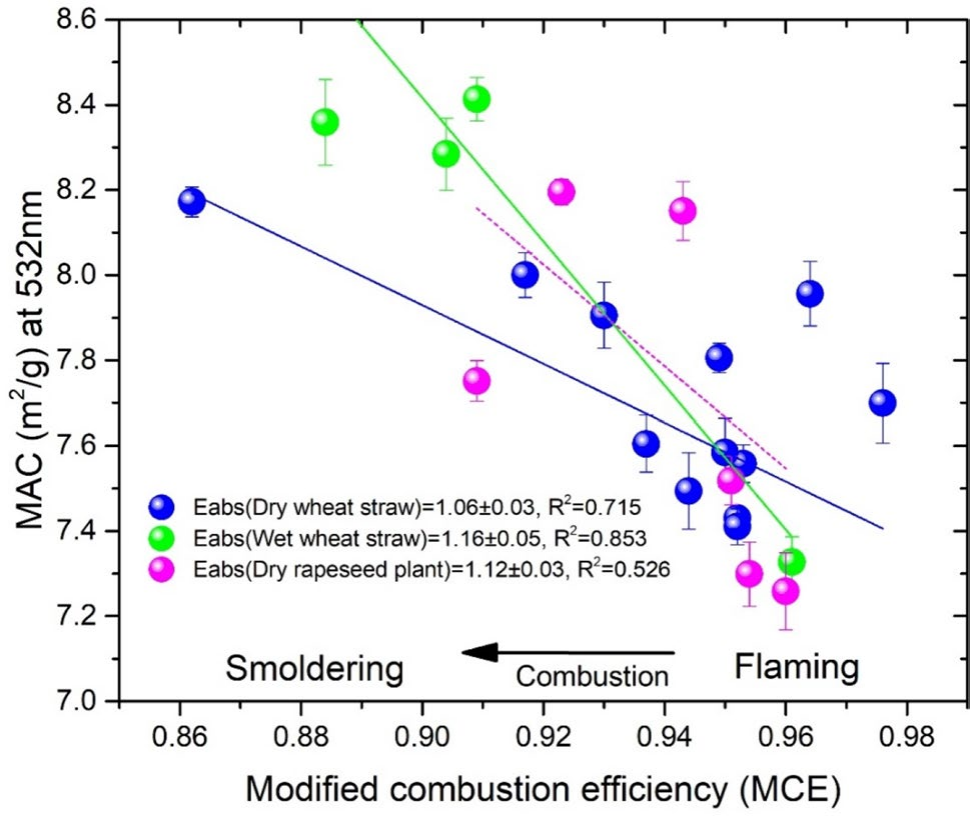
Variations of the mass absorption cross sections (MAC) with modified combustion efficiency (MCE) at 532 nm for different types and humidity levels of biomass sources, including dry wheat straw (blue), wet wheat straw (green), and dry rapeseed plants (magenta). The absorption enhancement is estimated according to the absorption ratio of BC-containing aerosols emitted from MCE = 0.86 to MCE = 0.98.

The effect of the biomass source on the MCE-dependent MAC of the BC-containing aerosols was further investigated, as shown in Fig. 3. The distributions of the MED and S/C ratio for 22 samples of three types of biomass sources were measured (Table S1), including dry wheat straw, wet wheat straw, and dry rapeseed plants. The averaged MCEs show no significant differences for the combustion of dry wheat straw (0.86–0.98), wet wheat straw (0.88–0.96), or dry rapeseed plants (0.91–0.96). The light absorption enhancement of carbonaceous aerosols was estimated by simple linear fits of the absorption ratio of the BC-containing aerosols emitted over 0.86–0.98 MCE. The *E*_*abs*_ obtained from the dry wheat straw was 1.06 ± 0.03 from the flaming-dominated states with an MCE of 0.98 to the smouldering-dominated states with an MCE of 0.86. Wet conditions may lead to larger values of *E*_*abs*_ with wheat straw, which implies that high relative humidity conditions may be important and should be included in the assessment of light absorption. A possible reason for this result is that the wet biomass was unfavourable for the production of BC particles in the flaming-dominated burning states and led to a smaller MAC, and the hygroscopic behaviour may generate a slightly thicker non-BC coating in the smouldering-dominated states, thus leading to a lager MAC. These discrepancies in light absorption suggest that the measured *E*_*abs*_ of biomass burning in the ambient environment may be influenced by regional planting areas and weather conditions.

**Figure 4:**
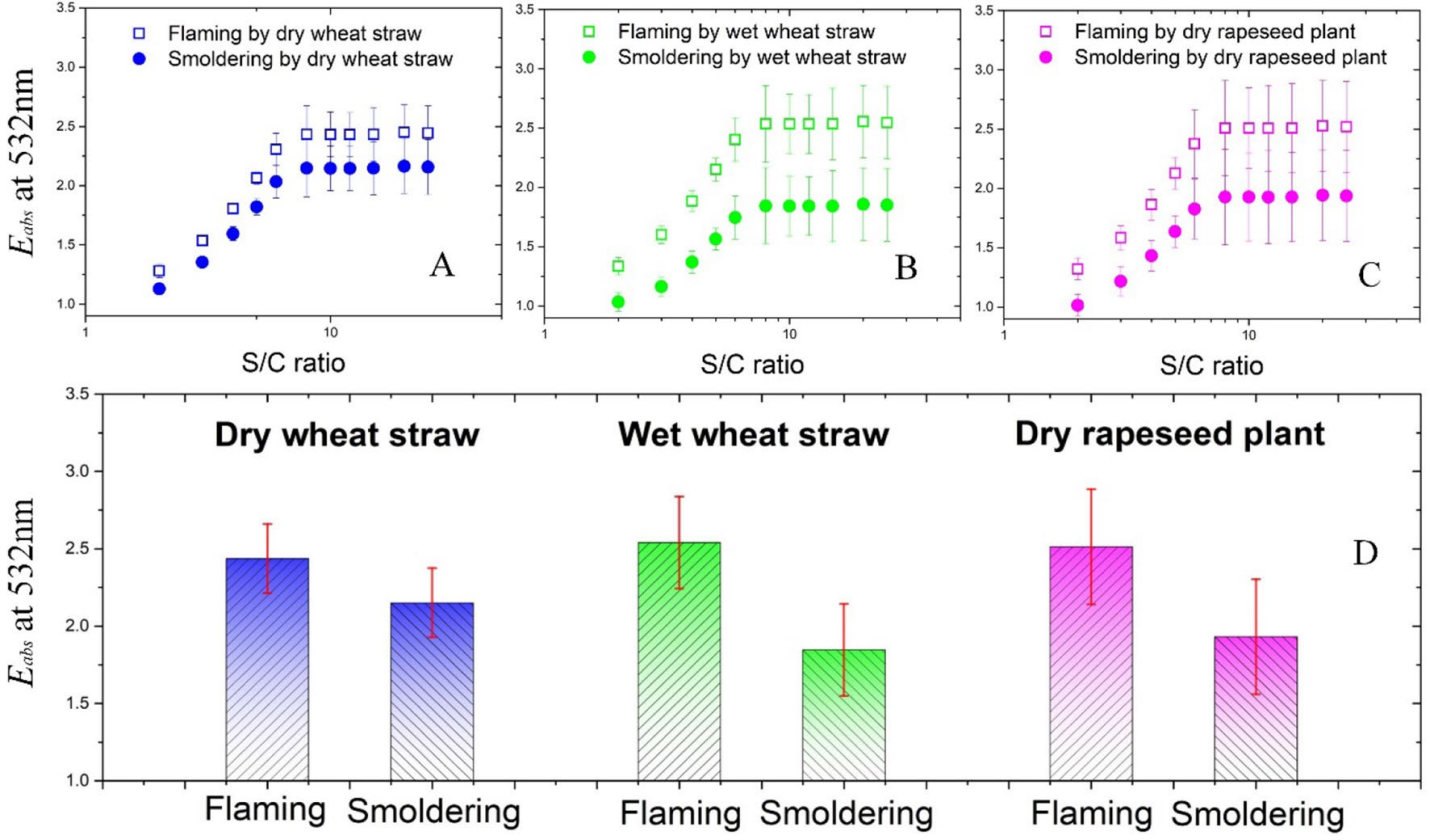
Sensitivity of the light absorption enhancement (*E*_*abs*_) estimated by the initial absorption of carbonaceous aerosols freshly emitted from different biomass burning states. (**A**) The variations of *E*_*abs*_ with an increased shell-core (S/C) ratio for dry wheat straw, where the initial absorption of the freshly emitted carbonaceous aerosols is assumed to be by the production of flaming-dominated combustion (hollow squares) and smouldering combustion (solid circles); (**B**) and (**C**) are similar to (**A**) but include aerosols emitted from the burning of wet wheat straw and dry rapeseed plants, respectively; (**D**) Simulated *E*_*abs*_ of fully aged BC aerosols for different burning states and sources.

Figure 4 demonstrates that the estimation of BC absorption enhancement is strongly affected by freshly emitted carbonaceous aerosols from different biomass burning states. The MAC of carbonaceous aerosols freshly emitted from flaming-dominated and smouldering-dominated combustion types were used for the estimates of the initial absorption. The variation in *E*_*abs*_ caused by the combustion states was investigated according to their different types and the humidity levels of the biomass sources, including dry wheat straw (Fig. 4A), wet wheat straw (Fig. 4B), and dry rapeseed plants (Fig. 4C). The simulated *E*_*abs*_ rises rapidly for BC-containing aerosols with S/C ratio smaller than ~ 6, and further coating leads to a stable value of *E*_*abs*_. This result agrees with previous measurements and simulations^44–47^. In the rapidly rising stage of *E*_*abs*_ versus the S/C ratio, the effects of different combustion states on *E*_*abs*_ are remarkable and thus significantly influence any estimates of *E*_*abs*_. For wet wheat straw and dry rapeseed plants, the *E*_*abs*_ values of BC-containing aerosols freshly emitted from the smouldering-dominated states are limited to ~ 1.2, but are up to ~ 1.6 for flaming-dominated states, when the S/C ratio is ~ 3. The diversity in BC-containing aerosols from different combustion states in *E*_*abs*_ also varied for different types and humidity levels of biomass sources. The variation in *E*_*abs*_ of wheat straw under wet conditions is also obviously larger than that under dry conditions. Moreover, in the stable stage of *E*_*abs*_, the varied combustion states may also lead to large diversity. The *E*_*abs*_ values are ranged from ~ 1.5 to ~ 2.2 for the smouldering-dominated states and ranged from ~ 2.1 to ~ 2.8 for the flaming-dominated states. These simulated results suggest that estimates of *E*_*abs*_ related to ambient biomass burning would benefit from constraining the combustion states.

The freshly emitted states of carbonaceous aerosols may be different in modelling simulations and ambient observations, leading to uncertain estimations of *E*_*abs*_. In previous studies, bare BC particles without any non-BC coatings were used as the freshly emitted state for *E*_*abs*_ estimations, but this particle morphology is not dominant in the real atmosphere. China et al. showed that only ~ 4% of the individual freshly emitted wildfire carbonaceous particles are bare^18^. Compared to the lifetime (1 day to 1 week) of BC aerosols in the atmosphere^10^, the atmospheric aging time of BC emissions from different biomass burning states are sufficiently short (< 10 s) in this study and can thus be considered to represent freshly emitted states for estimating *E*_*abs*_ in the ambient environment^13^. Figure 4D shows that the *E*_*abs*_ between the fully aged and freshly emitted BC aerosols vary significantly with the different combustion states of biomass burning. Compared to the previous absorption measurements, the values of *E*_*abs*_ are ~ 2.6 by assuming that the BC aerosols are freshly emitted from flaming-dominated combustion^46–49^ and decrease to ~ 1.8 for smouldering-dominated combustion^44,45,50–52^. From recent laboratory observations, the *E*_*abs*_ of fresh biomass burning emissions are estimated to be 1.7–1.9^53^ and this result corresponds with the simulation of BC particles emitted from smouldering-dominated combustion. The ambient measurements may also be influenced by transport and removal rates^10^, thus, the morphologies and mixing states of freshly emitted carbonaceous aerosols require further investigation for the initial parameterization of *E*_*abs*_ in different regions and seasons.

The discrepancies between field observations and theoretical modelling of BC absorption enhancement can be reduced by better understanding the initial and final states of carbonaceous aerosols in the ambient environment. The small values of *E*_*abs*_ measured in field observations may be due to the uncertain reference to bare BC in the freshly emitted state, and it is thus, important to provide a suitable synchronization of the field observations and theoretical modelling on the particle morphologies and mixing states of freshly emitted carbonaceous aerosols. To improve climate model predictions, it is imperative to better understand both the mass absorption cross-section of the freshly emitted BC aerosols and the enhancement of BC absorption after atmospheric aging. Constraining the freshly emitted states of carbonaceous aerosols in the estimates of *E*_*abs*_ should be helpful for the assessment of aerosol radiative forcing caused by biomass burning.

## Discussion and conclusions

Our analysis shows that the exact description of freshly emitted carbonaceous aerosols plays a key role in the estimates of the light absorption enhancement. These different combustion states could dramatically influence the absorption of carbonaceous aerosols freshly emitted from burning biomass, leading to varied estimates of *E*_*abs*_ across a wide range of flaming-dominated and smouldering-dominated combustion states. The parameterizations of the morphologies and mixing states of the ambient carbonaceous aerosols freshly emitted from burned biomass may provide a possible reconciliation of the discrepancies between ambient observations and model simulations. The small *E*_*abs*_ values measured by field observations may be initialized by freshly emitted BC aerosols dominated by a coated particle morphology rather than those with a bare morphology. The major uncertainties of these simulations come from particle size, morphology, components and mixing states of BC-containing aerosols^54^. In this study, the measured S/C ratio is limited by the instrument owing to their weak scattering signal, thus, the BC-containing particles with relatively large BC cores (MED = 200 ± 10 nm) were used for the simulations of the entire aerosol ensembles emitted from different biomass burning states. More accurate instruments would be helpful in the future studies. Moreover, the type and humidity of the biomass sources may lead to significant uncertainty regarding the mass absorption cross-section of the freshly emitted BC aerosols, which vary with different agriculture areas and weather conditions and should be considered in regional and global climate models. Thus, the initial states of freshly emitted carbonaceous aerosols should be further studied by investigating wildfires in regions with different types of land cover, such as forests and grassland^55^. Furthermore, the absorption of freshly emitted carbonaceous aerosols may also be affected by the co-emitted aerosol materials, such as brown carbon, organic carbon, and other light absorbing carbon aerosols^56–58^, a subject which also requires future research. Determining the initial states of carbonaceous aerosols freshly emitted from a burning biomass and their final states after atmospheric aging may provide exact parameterizations for climate modelling and would help reduce the uncertainty of the aerosol radiative forcing assessments.

## Supplementary information

Supplementary Information 1.

## References

[1] Ramanathan V, Carmichael G (2008). Global and regional climate changes due to black carbon. Nat. Geosci..

[2] Bond TC (2013). Bounding the role of black carbon in the climate system: A scientific assessment. J. Geophys. Res.-Atmos..

[3] Jacobson MZ (2001). Strong radiative heating due to the mixing state of black carbon in atmospheric aerosols. Nature.

[4] Mikhailov EF, Vlasenko SS, Podgorny IA, Ramanathan V, Corrigan CE (2006). Optical properties of soot–water drop agglomerates: An experimental study. J. Geophys. Res-Atmos..

[5] Cappa CD (2012). Radiative absorption enhancements due to the mixing state of atmospheric black carbon. Science.

[6] Jacobson MZ (2013). Comment on “radiative absorption enhancements due to the mixing state of atmospheric black carbon”. Science.

[7] Cappa CD (2013). Response to comment on "Radiative absorption enhancements due to the mixing state of atmospheric black carbon". Science.

[8] Ramana MV (2010). Warming influenced by the ratio of black carbon to sulphate and the black-carbon source. Nat. Geosci..

[9] Gustafsson Ö, Ramanathan V (2016). Convergence on climate warming by black carbon aerosols. Proc. Natl. Acad. Sci. USA.

[10] Boucher O (2016). Jury is still out on the radiative forcing by black carbon. Proc. Natl. Acad. Sci. USA.

[11] Wu Y (2018). Light absorption enhancement of black carbon aerosol constrained by particle morphology. Environ. Sci. Technol..

[12] McMeeking GR (2014). Impacts of nonrefractory material on light absorption by aerosols emitted from biomass burning. J. Geophys. Res-Atmos..

[13] Pan X (2017). Emission characteristics of refractory black carbon aerosols from fresh biomass burning: A perspective from laboratory experiments. Atmos. Chem. Phys..

[14] Inomata S (2015). Laboratory measurements of emission factors of nonmethane volatile organic compounds from burning of Chinese crop residues. J. Geophys. Res-Atmos..

[15] Kondo Y (2011). Emissions of black carbon, organic, and inorganic aerosols from biomass burning in North America and Asia in 2008. J. Geophys. Res-Atmos..

[16] May AA (2014). Aerosol emissions from prescribed fires in the United States: A synthesis of laboratory and aircraft measurements. J. Geophys. Res-Atmos..

[17] Adler G, Riziq AA, Erlick C, Rudich Y (2010). Effect of intrinsic organic carbon on the optical properties of fresh diesel soot. Proc. Natl. Acad. Sci. USA.

[18] China S, Mazzoleni C, Gorkowski K, Aiken AC, Dubey MK (2013). Morphology and mixing state of individual freshly emitted wildfire carbonaceous particles. Nat. Commun..

[19] Wang Y (2017). Fractal dimensions and mixing structures of soot particles during atmospheric processing. Environ. Sci. Technol. Lett..

[20] Zhang R (2015). Formation of urban fine particulate matter. Chem. Rev..

[21] Cappa CD (2019). Light absorption by ambient black and brown carbon and its dependence on black carbon coating state for two California USA cities in winter and summer. J. Geophys. Res. Atmos..

[22] Adachi K, Chung SH, Friedrich H, Buseck PR (2007). Fractal parameters of individual soot particles determined using electron tomography: Implications for optical properties. J. Geophys. Res-Atmos..

[23] Zhang R (2008). Variability in morphology, hygroscopicity, and optical properties of soot aerosols during atmospheric processing. Proc. Natl. Acad. Sci. USA..

[24] Bond TC, Bergstrom RW (2006). Light absorption by carbonaceous particles: An investigative review. Aerosol Sci. Technol..

[25] Kahnert M, Nousiainen T, Lindqvist H (2014). Review: Model particles in atmospheric optics. J. Quant. Spectrosc. Radiat. Transfer.

[26] Adachi K, Buseck PR (2008). Internally mixed soot, sulfates, and organic matter in aerosol particles from Mexico City. Atmos. Chem. Phys..

[27] Levin EJT (2010). Biomass burning smoke aerosol properties measured during Fire Laboratory at Missoula Experiments (FLAME). J. Geophys. Res-Atmos..

[28] Jarzembski MA, Norman ML, Fuller KA, Srivastava V, Cutten DR (2003). Complex refractive index of ammonium nitrate in the 2–20-μm spectral range. Appl. Opt..

[29] Chakrabarty RK (2010). Brown carbon in tar balls from smoldering biomass combustion. Atmos. Chem. Phys..

[30] Pósfai M, Buseck PR (2010). Nature and climate effects of individual tropospheric aerosol particles. Annu. Rev. Earth Planet Sci..

[31] Shamjad PM, Satish RV, Thamban NM, Rastogi N, Tripathi SN (2018). Absorbing refractive index and direct radiative forcing of atmospheric brown carbon over Gangetic Plain. ACS Earth Space Chem..

[32] Wu Y, Cheng T, Zheng L, Chen H (2016). Optical properties of the semi-external mixture composed of sulfate particle and different quantities of soot aggregates. J. Quant. Spectrosc. Radiat. Transfer.

[33] He C (2015). Variation of the radiative properties during black carbon aging: Theoretical and experimental intercomparison. Atmos. Chem. Phys..

[34] Wu Y, Cheng T, Zheng L, Chen H (2016). Black carbon radiative forcing at TOA decreased during aging. Sci. Rep..

[35] Mackowski DW, Mishchenko MI (2011). A multiple sphere T-matrix Fortran code for use on parallel computer clusters. J. Quant. Spectrosc. Radiat. Transfer.

[36] Mishchenko MI, Liu L, Cairns B, Mackowski DW (2014). Optics of water cloud droplets mixed with black-carbon aerosols. Opt. Lett..

[37] Liu D (2017). Black-carbon absorption enhancement in the atmosphere determined by particle mixing state. Nat. Geosci..

[38] Yon J (2014). Effects of multiple scattering on radiative properties of soot fractal aggregates. J. Quant. Spectrosc. Radiat. Transfer.

[39] Dastanpour R, Momenimovahed A, Thomson K, Olfert J, Rogak S (2017). Variation of the optical properties of soot as a function of particle mass. Carbon.

[40] Kahnert M, Kanngießer F (2020). Modelling optical properties of atmospheric black carbon aerosols. J. Quant. Spectrosc. Radiat. Transfer.

[41] Kahnert M (2017). Optical properties of black carbon aerosols encapsulated in a shell of sulfate: Comparison of the closed cell model with a coated aggregate model. Opt. Express.

[42] Cheng Y (2016). Light absorption by biomass burning source emissions. Atmos. Environ..

[43] Zangmeister CD (2018). Measured in-situ mass absorption spectra for nine forms of highly-absorbing carbonaceous aerosol. Carbon.

[44] Shiraiwa M, Kondo Y, Iwamoto T, Kita K (2010). Amplification of light absorption of black carbon by organic coating. Aerosol Sci. Techol..

[45] Saliba G (2016). Optical properties of black carbon in cookstove emissions coated with secondary organic aerosols: Measurements and modeling. Aerosol Sci. Technol..

[46] Peng J (2016). Markedly enhanced absorption and direct radiative forcing of black carbon under polluted urban environments. Proc. Natl. Acad. Sci. USA.

[47] Wu Y, Cheng T, Zheng L, Chen H (2017). Sensitivity of mixing states on optical properties of fresh secondary organic carbon aerosols. J. Quant. Spectrosc. Radiat. Transfer J..

[48] Cui X (2016). Radiative absorption enhancement from coatings on black carbon aerosols. Sci. Total Environ..

[49] You R, Radney JG, Zachariah MR, Zangmeister CD (2016). Measured wavelength-dependent absorption enhancement of internally mixed black carbon with absorbing and nonabsorbing materials. Environ. Sci. Technol..

[50] Schnaiter M (2005). Absorption amplification of black carbon internally mixed with secondary organic aerosol. J. Geophys. Res-Atmos..

[51] Bueno PA (2011). Photoacoustic measurements of amplification of the absorption cross section for coated soot aerosols. Aerosol Sci. Technol..

[52] Liu S (2015). Enhanced light absorption by mixed source black and brown carbon particles in UK winter. Nat. Commun..

[53] Wang Q (2018). Enhanced light absorption due to the mixing state of black carbon in fresh biomass burning emissions. Atmos. Environ..

[54] Liu F (2020). Review of recent literature on the light absorption properties of black carbon: Refractive index, mass absorption cross section, and absorption function. Aerosol Sci. Technol..

[55] Chakrabarty RK (2014). Soot superaggregates from flaming wildfires and their direct radiative forcing. Sci. Rep..

[56] Washenfelder RA (2015). Biomass burning dominates brown carbon absorption in the rural southeastern United States. Geophys. Res. Lett..

[57] Healy RM (2015). Light-absorbing properties of ambient black carbon and brown carbon from fossil fuel and biomass burning sources. J. Geophys. Res-Atmos..

[58] Sumlin BJ (2018). UV-Vis-IR spectral complex refractive indices and optical properties of brown carbon aerosol from biomass burning. J. Quant. Spectrosc. Radiat. Transfer.

