# Supplementary material for: The role of biomass burning states in light absorption enhancement of carbonaceous aerosols
Source: Sci Rep. 2020 Jul 30;10:12829. doi: 10.1038/s41598-020-69611-w (PMC7393073; doi:10.1038/s41598-020-69611-w)
Supplement: Supplementary file 1 — Supplementary Information 1. [file 41598_2020_69611_MOESM1_ESM.docx]

**Supporting Information of “The role of biomass burning states in light absorption enhancement of carbonaceous aerosols”**

Yu Wu^1^, Tianhai Cheng^1*^, Xiaole Pan ^2^, Lijuan Zheng ^3^, Shuaiyi Shi ^1^, Hang Liu ^2^

^1^ State Key Laboratory of Remote Sensing Science, Aerospace Information Research Institute, Chinese Academy of Sciences, No.9 Dengzhuangnan Road, Haidian District, Beijing, 100094, China.

^2^State Key Laboratory of Atmospheric Boundary Layer Physics and Atmospheric Chemistry, Institute of Atmospheric Physics, Chinese Academy of Sciences, No.40 Huayanli, Chaoyang District, Beijing, 100029, China.

^3^Land Satellite Remote Sensing Application Center, Ministry of Natural Resources of China, No.1 Baishengcun, Haidian District, Beijing, 100048, China.

*Correspondence to: Tianhai Cheng, [chength@radi.ac.cn](mailto:chength@radi.ac.cn).

## S1 Descriptions of biomass burning experiments

The biomass burning experiments, were performed in a combustion chamber in a laboratory environment and were conducted using dry wheat straw, wet wheat straw and dry rapeseed plants, as shown in Figure S1. The samples of wheat straw and rapeseed plants were collected from agricultural area of East China. All samples were stored in sealed plastic bags to preserve their initial states. Eighteen samples were directly burned in the chamber (referred to as “dry”), and four samples (referred to as “wet”) were placed in humid conditions (RH>99%) for 30 min. The samples were placed on an aluminum foil net rack and ignited by a butane-fuel lighter. The inlets of the sampling tube were ~40cm away from the burning place and a dilution system was used to avoid the high concentration loading of particles and gases produced by burning. A 50 cm long, 1/4-inch flexible conductive silicon tube were used for aerosol sampling and a polytetrafluoroethylene (PTFE) tube was used for gases sampling. The residence time was very short (~6 s) to minimizing the aging of aerosols in the tube [1-2].

Biomass burning leads to the production of BC, organic matter and other aerosol components, the particle size, morphology, and mixing states of which vary with their combustion states. Most carbon substances are converted to carbon dioxide and BC particles in flaming-dominated combustion, while further smoldering-dominated combustion predominantly emits carbon monoxide and organics [1]. The flaming and smoldering combustion stages were classified using the modified combustion efficiency (MCE), which was calculated on the basis of fire-integrated excess CO and CO_2_ mixing ratios, relative to their background values. It is defined as$\Delta\text{CO}_{2}/\left(\Delta\text{CO}_{2}+\Delta\text{CO}\right)$, indicating different combustion states in biomass burning. A MCE value >0.95 is normally regarded as flaming-dominant combustion, whereas MCE value <0.9 represents the smoldering-dominant combustion [3-4].

The mixing ratio of CO_2_ and CO was measured using a Li-7000 CO2 analyzer (Li-COR Inc.) and an ultrafast CO analyzer (model AL5002, Aero-Laser GmbH). A single particle soot photometer (SP2, Droplet Measurement Technologies Inc.) was used to measure the size distribution and shell-core ratio of BC. SP2 was well calibrated using the fullerene soot (stock 40971, lot: L20W054, Alfa Aesar, USA) and polystyrene sphere latex particles (PSL. JSR Corporation, Japan). The mass of a single BC particle can be directly measured by SP2. The diameter of BC core (*D_c_*) can be determined by assuming an ideal spherical structure and BC density of 1.8g/cm^3^. The size of entire BC-containing particle (*D_p_*) was retrieving from the leading-edge only (LEO) fit method [5]. The reliability of LEO method was examined by measuring laboratory generated coated particles of known shell-core ratio values. Measured shell-core (S/C) ratios showed good relationship with the real S/C values with a high correlation coefficient (r^2^=0.9). The uncertainty of the measured S/C values was determined to be 14%.

## S2 Sensitivity of optical simulations

In the simulations, the 2-D distributions of volume-equivalent BC core diameter and S/C ratio were designed for the optical calculations of the BC aerosol ensembles. In each case, the MCE values, the distributions of the volume-equivalent BC core diameter and the peak values of the S/C ratio were measured. In the simulations, the peaks of the S/C ratio were assumed to be mono-disperse for the varied volume-equivalent BC core diameter in each cases. Figure S2 shows that the deviations of mass absorption cross sections (MAC) of BC-containing aerosols between the poly-disperse and mono-disperse S/C ratios are limited to be less than 1.5%. Therefore, the absorption simulations of BC-containing aerosols with the distributions of S/C ratios can be calculated by their peaks of the distributions.

### S2.1 Effect of the BC fractal dimension

The fractal dimensions of BC particles are sensitive and important for their optical properties, and this morphological parameter tends to be weakened by mixing with larger non-BC components [6-7]. The mode mass equivalent diameter of BC cores ranged from 50 to 350 nm, which include most BC particles. The effect of the BC fractal dimensions on the absorption properties of BC particles freshly emitted from different combustion states was investigated. The absorption simulations of BC-containing particles were related to the Shell/Core ratio, and the fractal dimensions of freshly emitted BC particles with bare morphology were varied from 1.8, 2.0, 2.2, to 2.4. The optical properties of the BC aerosols were integrated by the distributions of the particle sizes and the S/C ratios.

Figure S3 shows that the MAC is slightly influenced by the fractal dimensions of bare BC particles. Larger fractal dimensions of bare BC particles indicate more compact morphologies of fractal aggregates. The refractive index of a BC component was assumed to be 1.95+0.79i in the visible and infrared range [8]. Figure S4 indicates that the trends of the simulated values of BC aerosols are consistent with the measurements by McMeeking et al. [9], and the refractive index of the non-BC component value of 1.55+10^-3^i is suggested for the absorption simulations of biomass burning. The MAC of BC aerosols freshly emitted from flaming-dominated combustion is ~7.3 for an assumed value of *D_f_*=1.8, and increases only ~1.5% if *D_f_*=2.4. The diversity of the MAC between flaming and smoldering states also slightly decreased with the more compact morphologies of bare BC particles. Previous studies have indicated that the absorption of freshly emitted BC particles may decrease with more compact particle morphologies [10]. A possible reason for this observation is that the loose structure of BC particles may have more monomers directly exposed to the incident light, while the lens effect is limited for bare and partly coated BC particles, because the light absorbing BC components predominate in these states.

### S2.2 Effect of non-BC refractive indices

The refractive indices of non-BC components may vary according to their aerosol types and environments [11-14]. Previous measurements indicated that the refractive indices of organic materials varied between 1.4 to 1.6 depending on several factors, including the parent hydrocarbon, oxidation chemistry, and the secondary organic aerosol (SOA) generation temperature. These assumed refractive indices also lie within the range of other aerosols, such as sulfates, nitrates, dust and sea salt. To investigate the effect of non-BC refractive indices on the BC-containing aerosols used in this study, the non-BC components were simulated with refractive index real part values of 1.4, 1.45, 1.5, 1.55 and 1.6, with the imaginary part assumed to be 0. The coatings are also assumed to be brown carbon, with the refractive index real part held constant at 1.55 and varying the imaginary parts with values of 0.001, 0.005, 0.01, 0.05 and 0.1.

Figure S5 demonstrates that the MAC of freshly emitted BC aerosols increases with the augmentation of the real parts of the non-BC refractive indices because of the intensified lens effect caused by mixing more non-BC components. The MAC of BC aerosols freshly emitted from flaming combustion is estimated to be ~7.1 m^2^/g at 532 nm, when $\mathrm{Re}_{non-BC}$ is 1.4. This MAC increased to ~7.7 m^2^/g for the smouldering stages and increases to ~8.3 if $\mathrm{Re}_{non-BC}$ equals 1.6. Larger real refractive indices of non-BC components may distinctly intensify the lens effect by scattering more lights towards the light absorbing BC components and lead to larger mass absorption cross sections at the beginning of the interaction between freshly emitted BC and non-BC particles.

The coating with larger imaginary parts of refractive indices leads to stronger absorption of BC-containing particles, and also enlarges the diversity of MAC between flaming and somldering combustions. When $\mathrm{Im}_{non-BC}$ is 0.001, the MAC of BC aerosols freshly emitted from smoldering combustion is ~10% larger than those from flaming combustion, and this diversity increased to ~25% if $\mathrm{Im}_{non-BC}$ equals 0.1. The absorption of non-BC coating also amplified the lens effect of BC-containing particles, and it should be noted in the optical simulations.

As shown in Figure S9, different simulations of the dependence on MCE with possible refractive index imaginary parts of non-BC components were performed for brown carbon mixing with BC particles; the used imaginary index values were 0.001, 0.01, 0.02, and 0.1, and the real part was held constant at 1.55. The variation of the BC absorption for different biomass burning states tends to become larger for the more absorbing non-BC components. The MAC of the freshly emitted BC aerosols from the smouldering-dominated combustion states were ~1.09, ~1.12, ~1.14, and ~1.21 times larger than those from flaming-dominated combustion at 532 nm when the refractive index imaginary parts of the non-BC components were 0.001, 0.01, 0.02, and 0.1, respectively.

### S2.3 Effect of the non-BC density of non-BC components

The uncertainty of absorption caused by the varied density of non-absorption particle is investigated from 1.0 to 1.2 g/cm^3^, as shown in Figure S6. The relative deviations of MAC between different densities of non-BC particles are limited to ~0.3% for the flaming-dominated combustion states, and ~0.5% for the smoldering-dominated states. As a result, the relative deviations of MAC between the flaming and smoldering states is varied from ~1% due to the uncertainty of the density of non-BC particle. The effect of density of non-absorption particle on absorption of BC aerosol ensembles freshly emitted from biomass burning is limited.

### S2.4 MAC varied with wavelength by the aggregate model

Figure S7 shows the MAC of BC aerosols at different combustion stages in the visible and near-infrared range, namely, 532, 670, 865, and 1064nm. Figure S8 shows the single scattering albedo and absorption Ångström exponent of the sampled cases. The absorption properties of BC aerosols with different particle sizes and S/C ratios are simulated and integrated by the measured distribution of particle size and S/C ratio at different combustion stages. The MAC of the freshly emitted BC aerosols is ~7.5 and ~8.3 in the flaming and smoldering combustion stages, respectively. The refractive indices of BC and non-BC particles are assumed to be 1.95+0.79i and 1.55+10^-3^i, respectively. This result agreed with the previous suggestion of 7.5±1.2m^2^/g according to a review of measurements [15]. Previous simulations showed that the MAC of bare BC is 6.0-6.5 m^2^/g [16-17], but this study illustrated that the measured freshly emitted BC may be not dominated by bare particle morphology. The diversity of MAC in freshly emitted BC aerosols between the flaming and smoldering states is from ~10%-15% in the visible and near-infrared range, increasing with the incident wavelength. The qualified BC absorption is related to the exact combustion stages and has important implications for assessing the impacts of BC on climate.

## Supplementary Figures


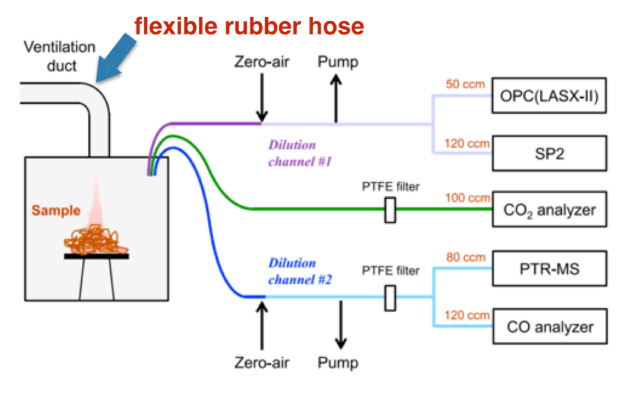


Figure S1 A schematic diagram of the laboratory biomass burning experiment.


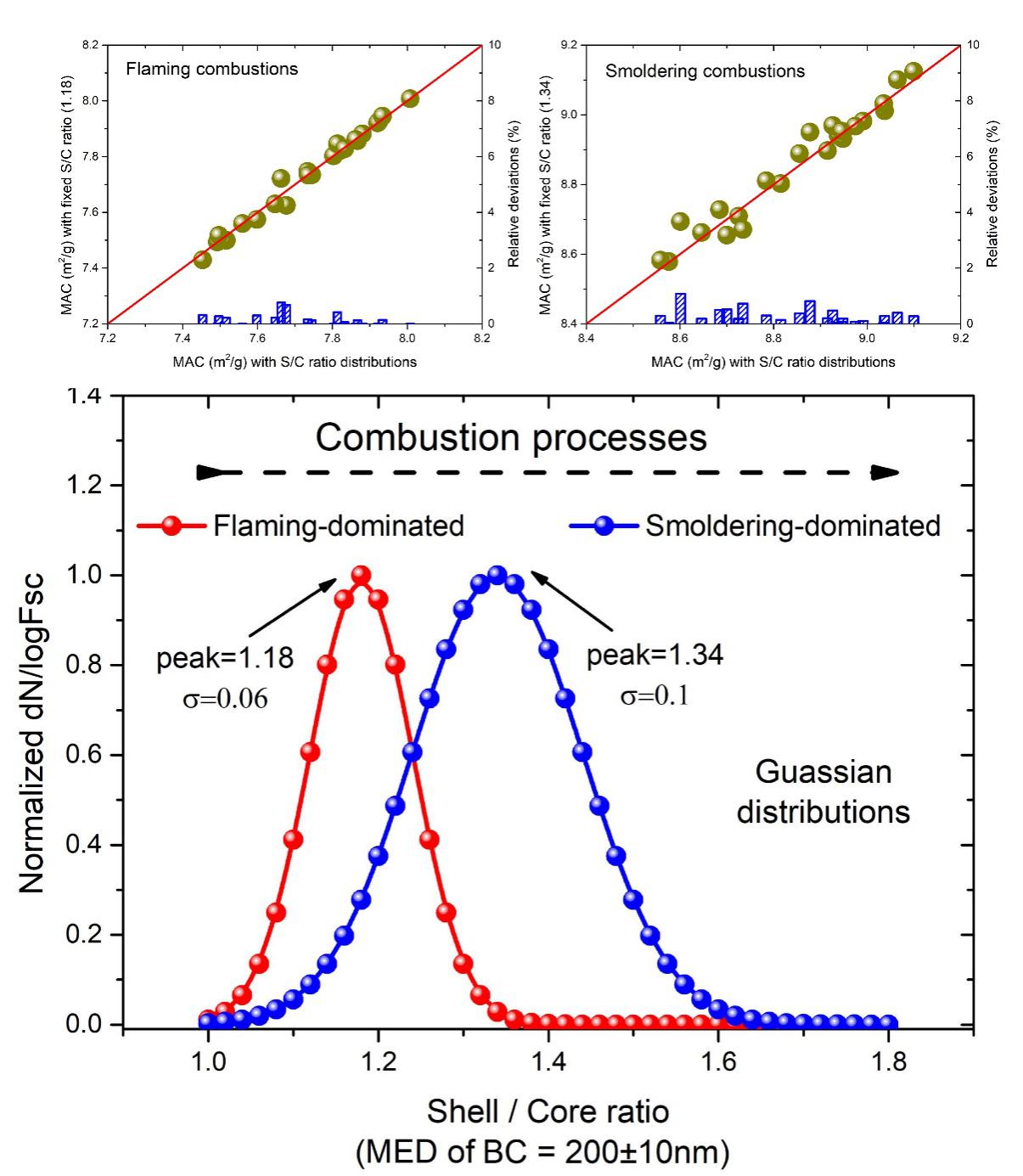


Figure S2 Distribution of the shell-core (S/C) ratio of the flaming-dominated and smouldering-dominated combustion states (bottom). The mass absorption cross sections (MAC) of the BC-containing aerosols were calculated and integrated using the distributions of the S/C ratio (X-axis) and the single peak values (Y-axis) for the flaming (top-left) and smouldering (top-right) combustion states, respectively. The blue bars are the relative deviations of MAC between the simulations by the distributions of the S/C ratio and the single peak values.


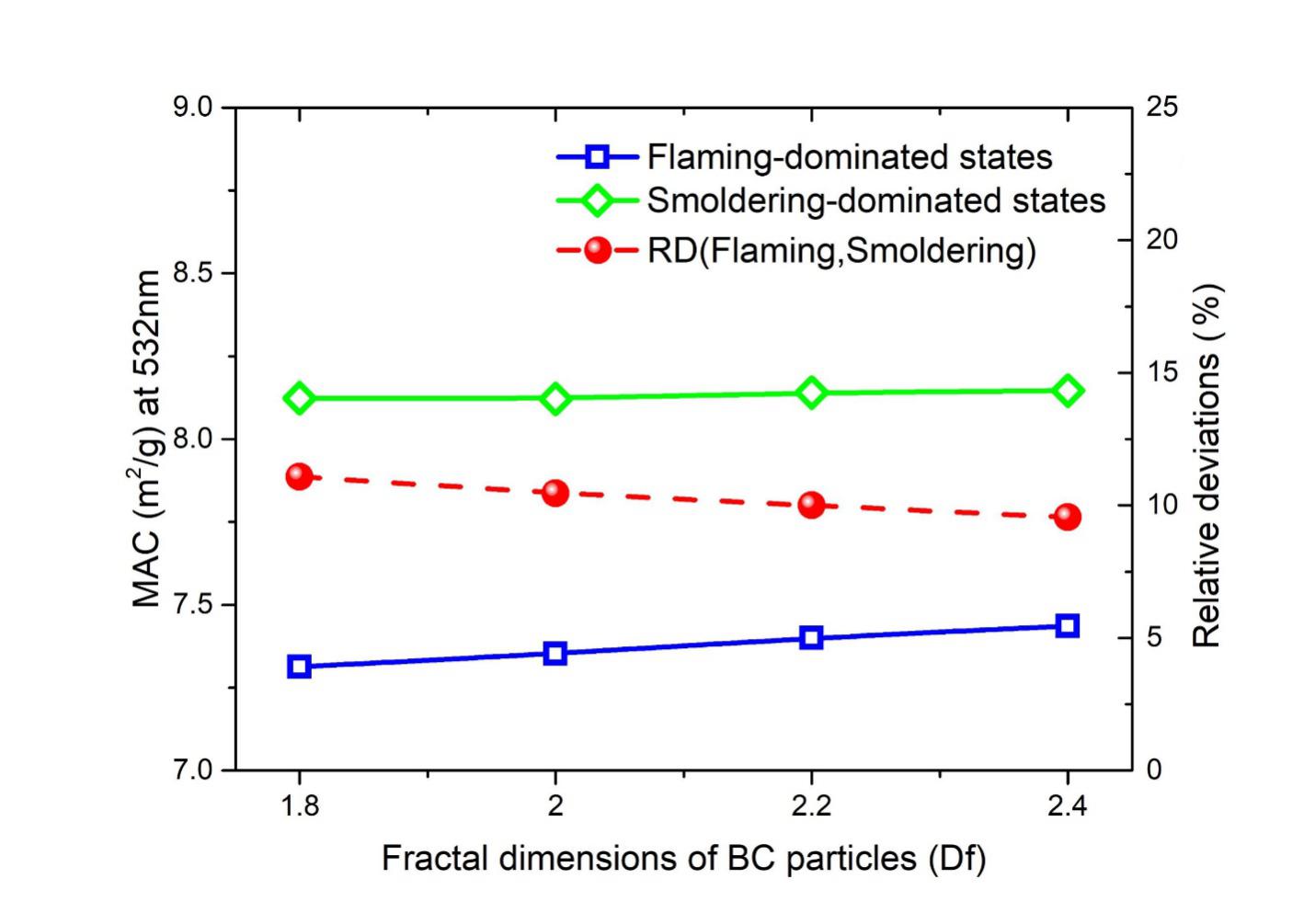


Figure S3 Sensitivity of the BC fractal dimensions (1.8–2.4) to the mass absorption cross section (MAC) of the BC aerosols at different combustion stages (left-axis), including the flaming-dominated (hollow-squares) and smouldering-dominated (hollow-rhombuses) states, and the relative deviations (solid-spheres) between the MAC of the flaming and smouldering stages (right-axis).


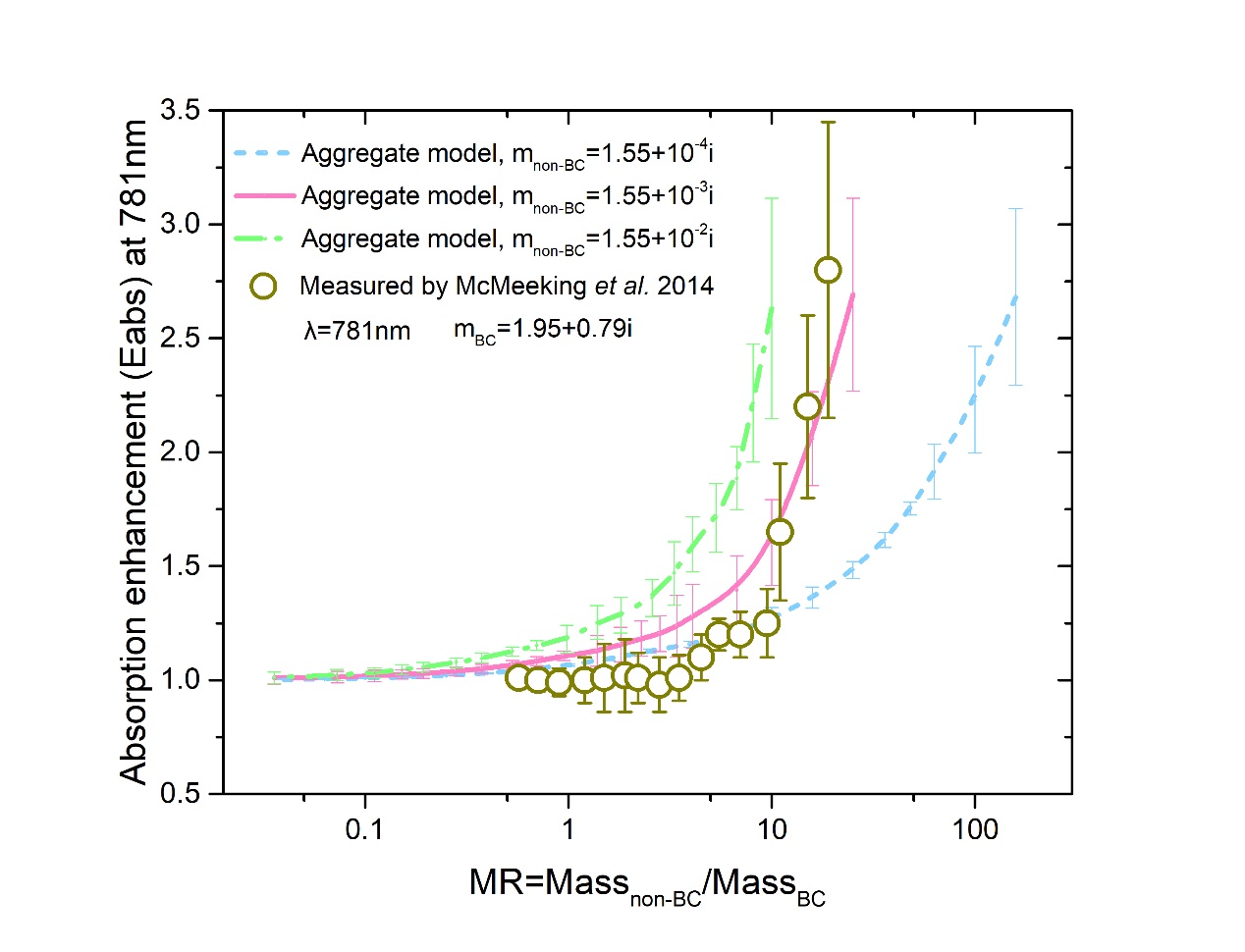


Figure S4 Comparison of BC absorption enhancement (yellow circle) measured by McMeeking et al. (2014) and typical simulations with different non-BC coatings, at a wavelength of 781 nm. The refractive index of the BC particles is assumed to be 1.95 + 0.79i. The real refractive indices of the non-BC coatings are held constant at 1.55 and their imaginary refractive indices were 10^-2^ (green line), 10^-3^ (pink line), and 10^-4^ (blue line).


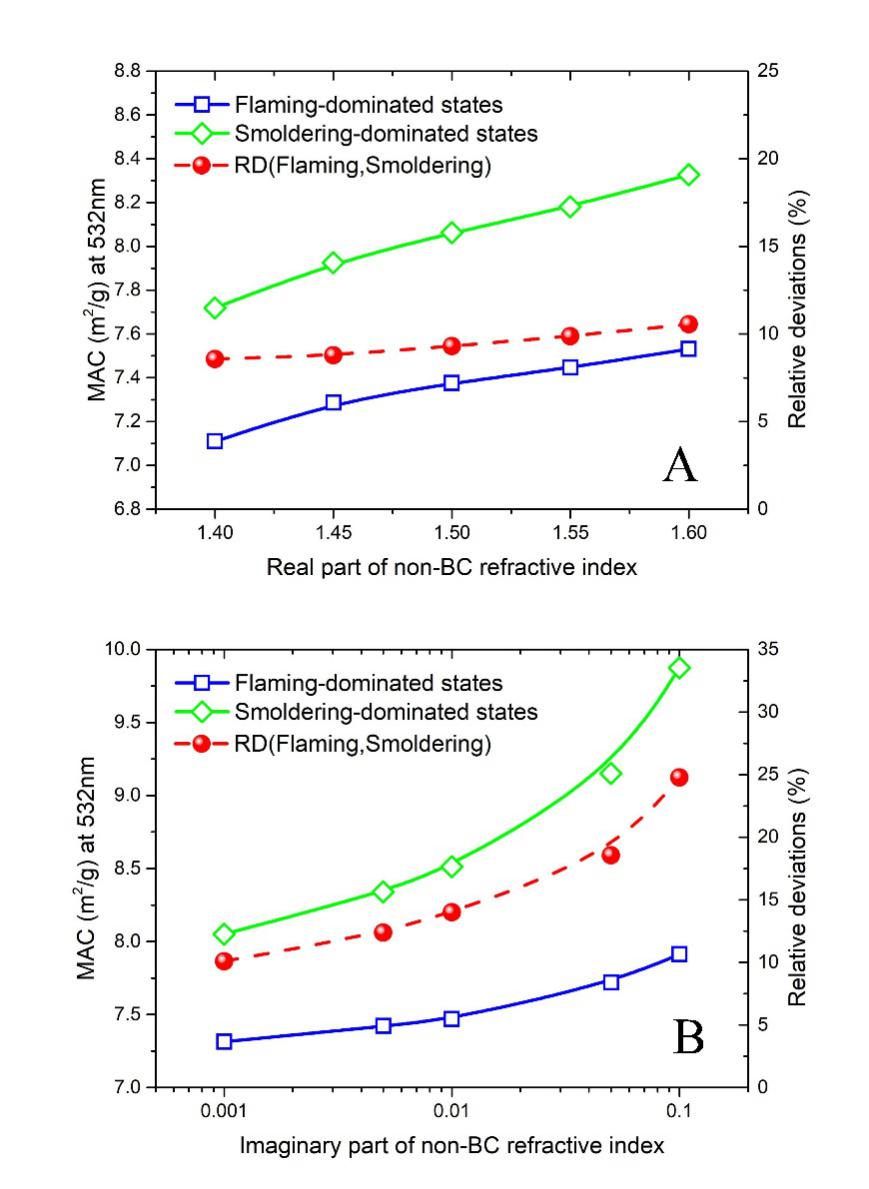


Figure S5 Sensitivity of non-BC refractive indices to the mass absorption cross section (MAC) of BC aerosols at different combustion stages (left-axis), including the flaming-dominated (blue hollow square) and smouldering-dominated (green hollow rhombus) states, and the relative deviations (red solid sphere) between the MAC of the flaming and smouldering stages (right-axis). Figure S5A shows typical sulphate and nitrate components, presents the real part of their refractive indices over the range of 1.4–1.6, with the imaginary part assumed to be 0. Figure S5B illustrates organic components, the imaginary parts of their refractive indices are over the range of 0–0.1, while their real parts are assumed to be 1.55, possibly due to brown carbon.


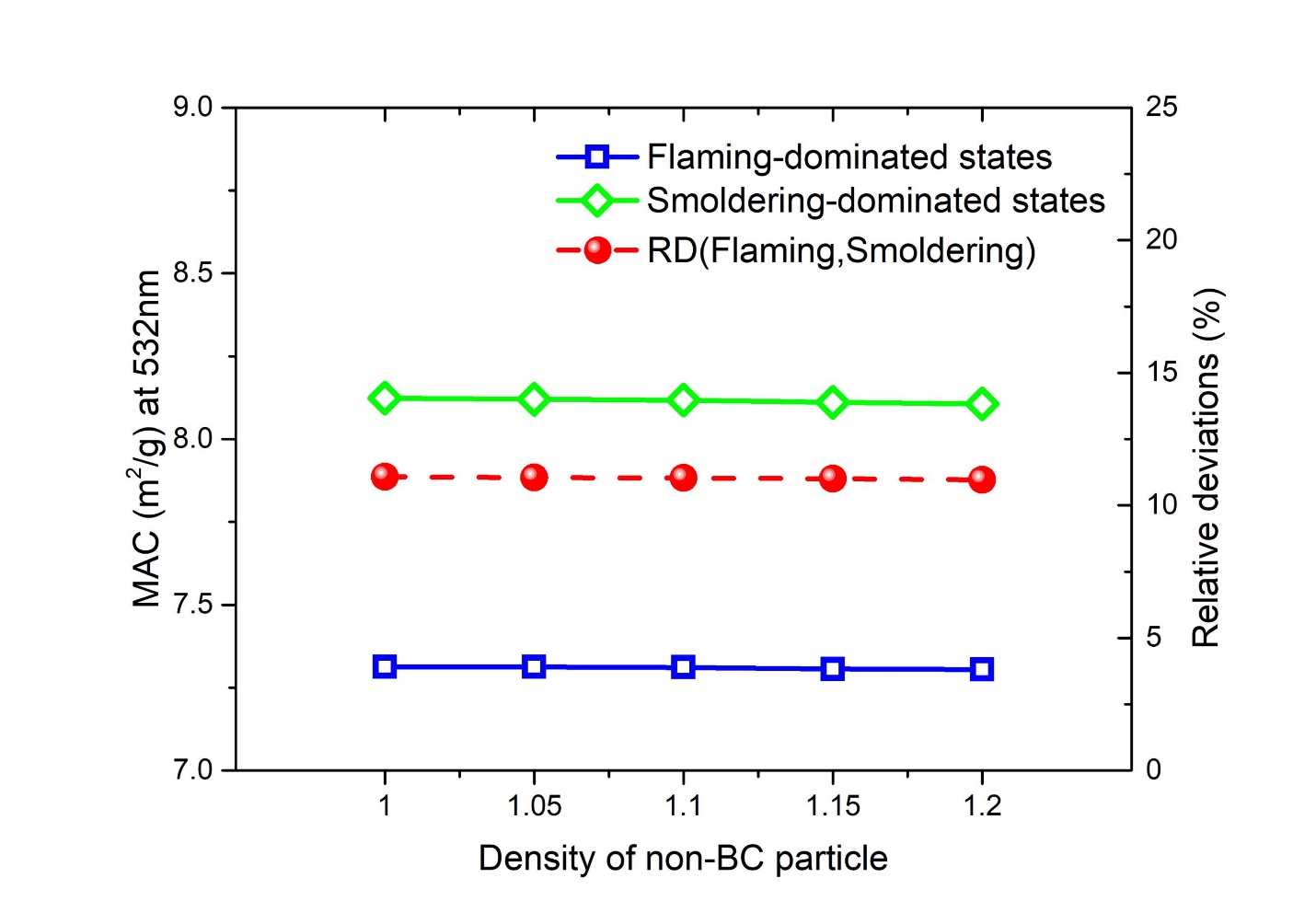


Figure S6 Sensitivity of the density of non-BC components in the individual BC-containing particles (1.0–1.2g/m^3^) to the mass absorption cross section (MAC) of BC aerosols at different combustion stages (left-axis), including the flaming-dominated (hollow-square) and smouldering-dominated (hollow-rhombus) states, and the relative deviations (solid-sphere) between the MAC of the flaming and smouldering stages (right-axis).


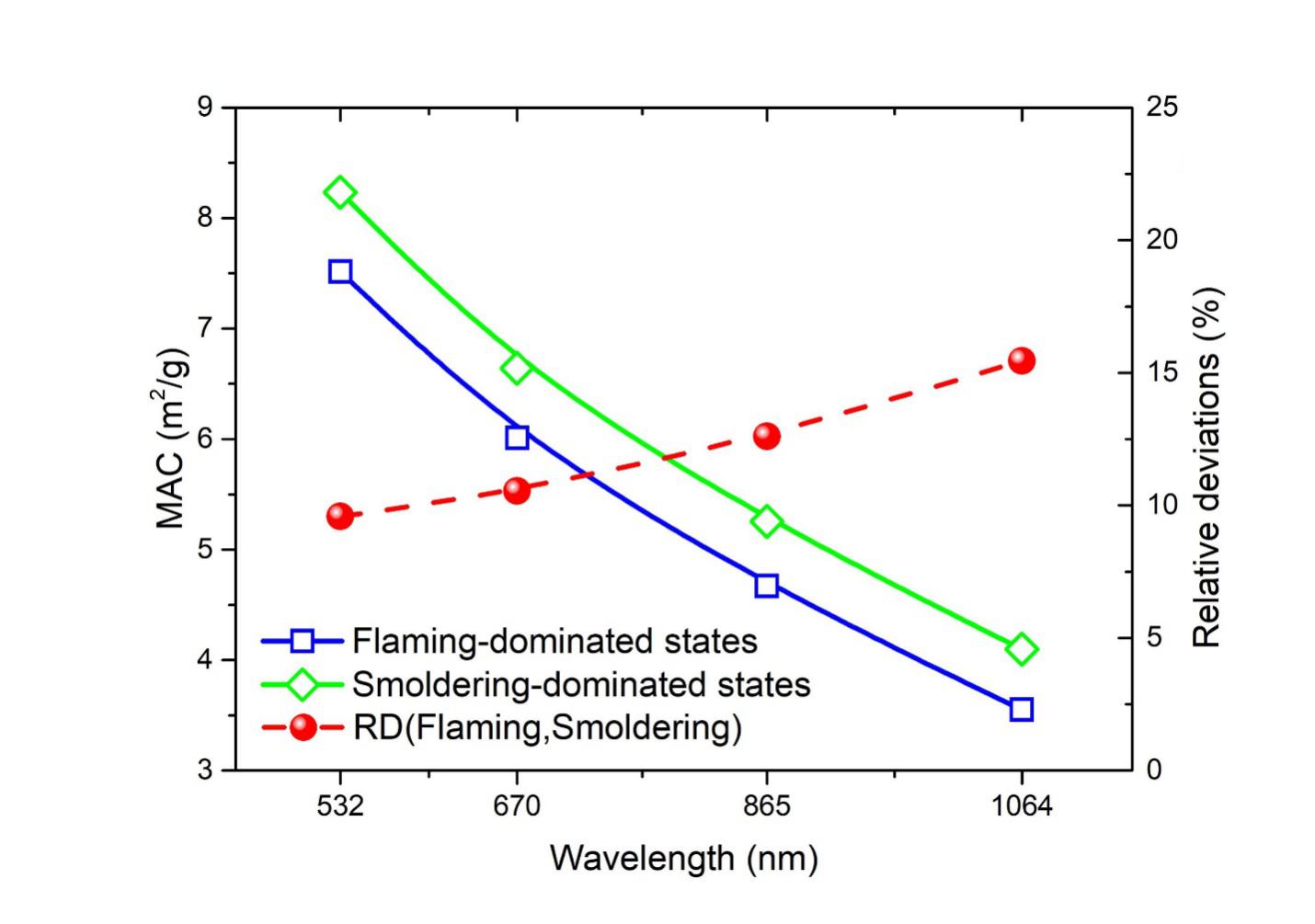


Figure S7 The mass absorption cross section (MAC) of BC aerosols at different combustion stages (left-axis) at visible and near-infrared wavelengths, including the flaming-dominated (hollow-square) and smouldering-dominated (hollow-rhombus) states, and the relative deviations (solid-sphere) of the MAC for the flaming and smouldering stages (right-axis).


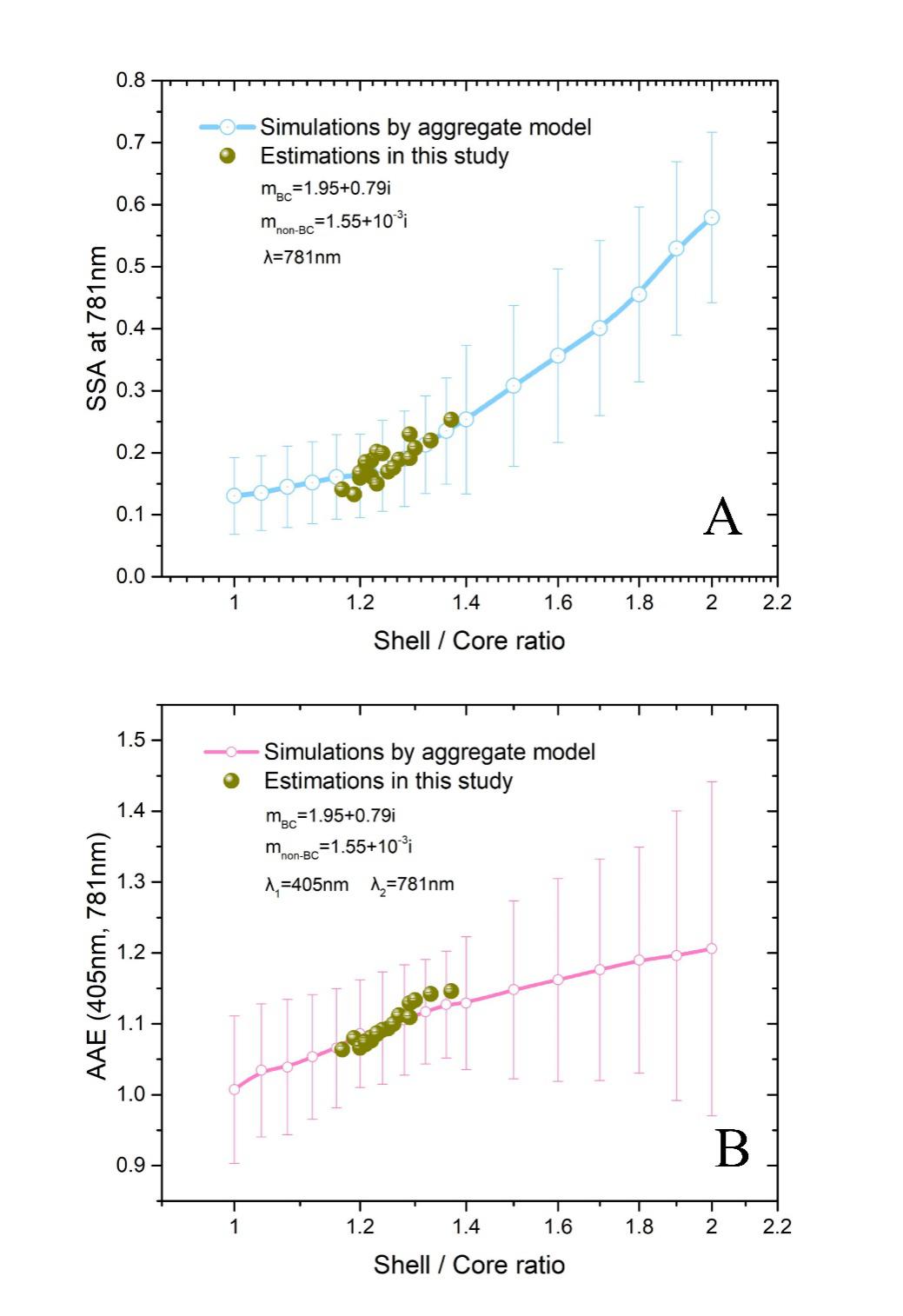


Figure S8 Variations of the optical properties of BC-containing aerosols with shell-core (S/C) ratios. (A) Single scattering albedo at 781 nm and (B) absorption Ångström exponent (AÅE) between 405 nm and 781 nm.


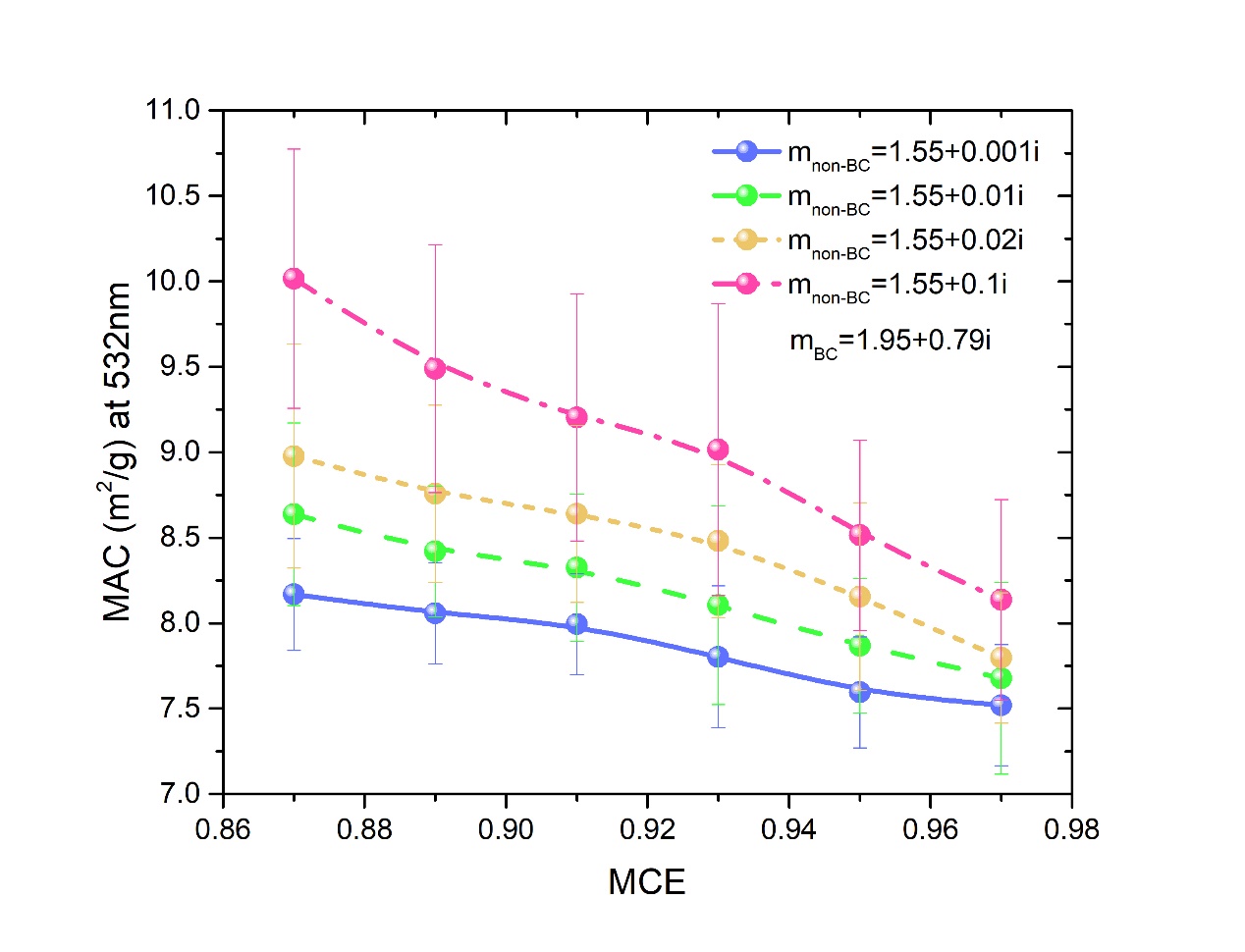


Figure S9 The mass absorption cross sections (MAC) of BC-containing aerosols mixed with different refractive index imaginary parts of the non-BC components at 532 nm predicted by the aggregate model is dependent upon the modified combustion efficiency (MCE) of the burned biomass, which indicates different combustion states. Generally, flaming-dominated combustion corresponds to a larger MCE (>0.95) and smouldering-dominated combustions correspond to a smaller MCE (<0.9).

## Supplementary Tables

Table S1 Physical properties of experimental samples.

| No. | Sample Type | MCE [10th, 90th] | MED | σ | S/C ratio |
| --- | --- | --- | --- | --- | --- |
| 1 | dry wheat straw | 0.964 [0.941, 0.991] | 215 | 0.278 | 1.19 |
| 2 | dry wheat straw | 0.93 [0.909, 0.982] | 188 | 0.378 | 1.25 |
| 3 | dry wheat straw | 0.952 [0.884, 0.973] | 152 | 0.365 | 1.21 |
| 4 | dry wheat straw | 0.949 [0.913, 0.999] | 187 | 0.365 | 1.22 |
| 5 | dry wheat straw | 0.953 [0.830, 0.987] | 160 | 0.351 | 1.21 |
| 6 | dry wheat straw | 0.976 [0.960, 0.994] | 191 | 0.315 | 1.17 |
| 7 | dry wheat straw | 0.917 [0.900, 0.987] | 187 | 0.385 | 1.27 |
| 8 | dry wheat straw | 0.944 [0.911, 0.979] | 148 | 0.358 | 1.23 |
| 9 | dry wheat straw | 0.862 [0.828, 0.920] | 152 | 0.365 | 1.37 |
| 10 | dry wheat straw | 0.937 [0.853, 0.988] | 148 | 0.351 | 1.24 |
| 11 | dry wheat straw | 0.95 [0.896, 0.976] | 163 | 0.378 | 1.22 |
| 12 | dry wheat straw | 0.952 [0.837, 0.964] | 160 | 0.405 | 1.21 |
| 13 | wet wheat straw | 0.909 [0.881, 0.999] | 196 | 0.285 | 1.29 |
| 14 | wet wheat straw | 0.904 [0.857, 0.999] | 177 | 0.307 | 1.3 |
| 15 | wet wheat straw | 0.961 [0.840, 0.988] | 148 | 0.372 | 1.2 |
| 16 | wet wheat straw | 0.884 [0.730, 0.999] | 181 | 0.344 | 1.33 |
| 17 | dry rapeseed plant | 0.943 [0.902, 0.999] | 204 | 0.255 | 1.23 |
| 18 | dry rapeseed plant | 0.923 [0.891, 0.999] | 189 | 0.285 | 1.26 |
| 19 | dry rapeseed plant | 0.909 [0.839, 0.947] | 137 | 0.329 | 1.29 |
| 20 | dry rapeseed plant | 0.951 [0.895, 0.976] | 155 | 0.344 | 1.21 |
| 21 | dry rapeseed plant | 0.96 [0.874, 0.985] | 142 | 0.365 | 1.2 |
| 22 | dry rapeseed plant | 0.954 [0.944, 0.994] | 144 | 0.385 | 1.21 |

## References

1. Pan, X. *et al*. Emission characteristics of refractory black carbon aerosols from fresh biomass burning: a perspective from laboratory experiments. *Atmos. Chem. Phys*. **17**, 13001-13016 (2017).
2. Inomata, S. *et al*. Laboratory measurements of emission factors of nonmethane volatile organic compounds from burning of Chinese crop residues. *J. Geophys. Res-Atmos.* **120**, 5237-5252 (2015).
3. Kondo, Y. *et al*. Emissions of black carbon, organic, and inorganic aerosols from biomass burning in North America and Asia in 2008. *J. Geophys. Res-Atmos*. **116**, D08204 (2011).
4. May, A. A. *et al*. Aerosol emissions from prescribed fires in the United States: A synthesis of laboratory and aircraft measurements. *J. Geophys. Res-Atmos*. **119**, 11826-11849 (2014).
5. Gao, R. S. *et al*. A novel method for estimating light-scattering properties of soot aerosols using a modified single-particle soot photometer. *Aerosol Sci. Tech.* **41**, 125-135 (2007).
6. T. Cheng, Y. Wu, H. Chen Effects of morphology on the radiative properties of internally mixed light absorbing carbon aerosols with different aging status. *Opt. Express* **22**, 15904-15917 (2014).
7. Liu, F., Yon, J. & Bescond A. On the radiative properties of soot aggregates-Part 2: Effects of coating. *J. Quant. Spectrosc. Radiat. Transfer* **172**, 134-145 (2016).
8. Bond, T. C. & Bergstrom R. W. Light absorption by carbonaceous particles: An investigative review. *Aerosol Sci. Technol.* **40**, 27-67 (2006).
9. McMeeking, G. R. *et al*. Impacts of nonrefractory material on light absorption by aerosols emitted from biomass burning. *J. Geophys. Res-Atmos.* **119**, 12272-12286 (2014).
10. Dong, J., Zhao, J. M. & Liu J. H. Morphological effects on the radiative properties of soot aerosols in different internally mixing states with sulfate. *J. Quant. Spectrosc. Radiat. Transfer* **165**, 43-55 (2015).
11. Jarzembski, M. A., Norman, M. L., Fuller, K. A., Srivastava, V. & Cutten, D. R. Complex refractive index of ammonium nitrate in the 2–20-μm spectral range. *Appl. Optics* **42**, 922-930 (2003).
12. Chakrabarty, R. K. *et al*. Brown carbon in tar balls from smoldering biomass combustion. *Atmos. Chem. Phys.* **10**, 6363-6370 (2010).
13. Pósfai, M. & Buseck P. R. Nature and climate effects of individual tropospheric aerosol particles. *Annu. Rev. Earth Pl. Sc.* **38**, 17-43 (2010).
14. Shamjad, P. M., Satish, R. V., Thamban, N. M., Rastogi, N. & Tripathi, S. N. Absorbing Refractive Index and Direct Radiative Forcing of Atmospheric Brown Carbon over Gangetic Plain. *ACS Earth Space Chem.* **2**, 31-37 (2018).
15. Ramana, M. V. *et al*. Warming influenced by the ratio of black carbon to sulphate and the black-carbon source. *Nat Geosci.*, **3**, 542-545 (2010).
16. Kahnert M. On the discrepancy between modeled and measured mass absorption cross sections of light absorbing carbon aerosols. *Aerosol Sci. Technol.* **44**, 453-460 (2010).
17. Wu, Y., Cheng, T., Zheng, L. & Chen, H. Effect of morphology on the optical properties of soot aggregated with spheroidal monomers. *J. Quant. Spectrosc. Radiat. Transfer* **168**, 158-169 (2016).
